# In Vivo Efficacy of a Broad-Spectrum Antiviral Combination Against Yellow Fever in a Hamster Model

**DOI:** 10.3390/pathogens14090925

**Published:** 2025-09-12

**Authors:** Abbie E. Weight, Hunter Stanger, Robert J. Geraghty, Laurent F. Bonnac, Justin G. Julander

**Affiliations:** 1 Institute for Antiviral Research, Utah State University, Logan, UT 84322, USA; 2 Center for Drug Design, College of Pharmacy, University of Minnesota, Minneapolis, MN 55455, USA

**Keywords:** broad-spectrum, drug combination, pandemic preparedness, favipiravir, yellow fever, flaviviruses, animal model

## Abstract

Yellow fever virus (YFV) recurrently causes severe outbreaks in tropical regions of South America and Africa and an average of 30 to 40 thousand deaths worldwide each year. An effective vaccine is available but the coverage of the population in countries at risk is not optimal. No antivirals are currently approved for YFV treatment. Herein, we describe the evaluation of 6-MMPr, a *de-novo*-purine-nucleotide biosynthesis inhibitor, as a potentiator for enhanced activity of the broad-spectrum antiviral drug favipiravir in a hamster model of yellow fever. Administration of 6-MMPr was well-tolerated and a combination of favipiravir and 6-MMPr did not cause overt toxicity as indicated by normal weight gain of treated hamsters. Treatment with a combination of a suboptimal dose of favipiravir with 6-MMPr was significantly more effective in improving survival, weight change and virus replication when compared with monotherapy. The initiation of treatment two days after virus challenge was also effective in improving survival when compared with monotherapy. Our results demonstrate the safety and efficacy of such a combination either as a preventive or delayed treatment.

## 1. Introduction

Yellow fever virus (YFV) is part of the RNA virus genus *Orthoflavivirus*, closely related to West Nile, St. Louis and Japanese encephalitis viruses. YFV is transmitted predominantly by *Aedes* and *Haemagogus* mosquitoes from tropical and subtropical regions of Africa and South America. The symptoms of yellow fever (YF) range from mild flu-like symptoms to severe hemorrhage and liver damage. While most cases are self-limited and resemble common viral infections, severe cases can be fatal, with mortality rates approaching 50%, underscoring the need for an effective treatment.

**Figure 1:**
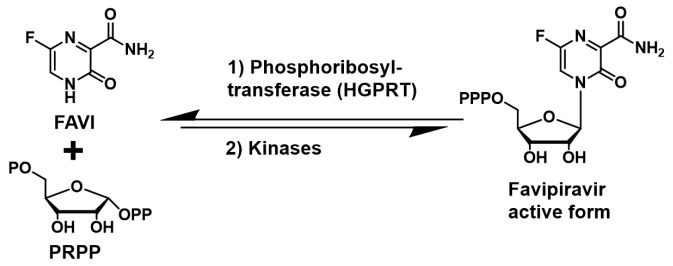
Favipiravir (FAVI) is converted in cells to its nucleoside triphosphate form.

Favipiravir (FAVI) is a broad-spectrum antiviral active against YFV in a hamster model [1]. Despite its exceptionally broad-spectrum antiviral effect against many viruses in vitro [1,2,3,4,5], FAVI demonstrated limited efficacy in clinical trials against Ebola [6,7], SARS-CoV-2 [8] and influenza viruses [9]. One of the possible reasons for FAVI’s limited efficacy is that it is a poor substrate for hypoxanthine-guanine phosphoribosyl-transferase (HGPRT) [10], the enzyme responsible for FAVI’s activation to its corresponding nucleotide via addition to the co-substrate phosphoribosyl pyrophosphate (PRPP) and phosphorylation by kinases (Figure 1).

**Figure 2:**
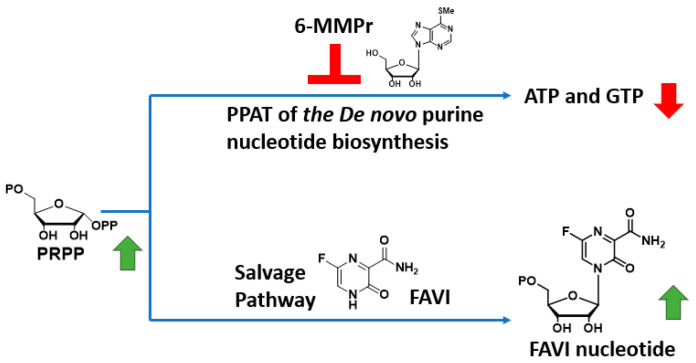
Mechanism behind FAVI/6-MMPr antiviral synergy. Green arrow: increase in concentration. Red arrow: decrease in concentration.

To circumvent this issue, we have developed a strategy to increase FAVI’s antiviral activity through a drug combination with a *de-novo*-purine-nucleotide biosynthesis inhibitor 6-methylmercaptopurine riboside (6-MMPr) (Figure 2) [11]. 6-MMPr inhibits the first enzyme of the *de-novo*-purine-nucleotide biosynthesis, phosphoribosyl-pyrophosphate amidotransferase (PPAT) [12] resulting in the accumulation of phosphoribosyl pyrophosphate (PRPP), the co-substrate required for the conversion of FAVI to its nucleotide form by HGPRT (Figure 2). Based on previous studies in patients, we hypothesized that a short term treatment with 6MMPr will not generate unwanted toxicity [13].

This combination was synergistic against major viruses such as influenza, dengue, SARS-CoV-2 and Zika viruses *in vitro* [11]. Despite these positive *in vitro* results, the safety of this drug combination and its translation to an efficient approach *in vivo* needed to be established. Herein we describe the evaluation of FAVI alone or combined with 6-MMPr in a YFV hamster model.

## 2. Materials and Methods

Animals: Specific pathogen-free female Syrian golden hamsters were obtained from Charles River Laboratories. Hamsters weighed between 80 and 90 g and were quarantined for at least three days prior to the initiation of experimental procedures. Groups included 3–10 animals. Group size was chosen based on power analysis run during model characterization studies. Hamsters were randomized after arrival at the LARC at USU.

Virus: A hamster-adapted (HA) Jimenez YFV strain was obtained from Robert Tesh (WRCEVA, UTMB, Galveston, TX, USA). This strain was originally isolated from a fatal human YF case in Panama and was passaged 10 times through hamsters for adaptation. We passaged the virus one additional time to prepare a challenge stock. Animals were challenged by bilateral intraperitoneal (IP) injections with 40 CCID_50_ of Jimenez HA YFV in a total volume of 0.2 mL.

Test agents: Favipiravir (FAVI) was purchased from Ambeed (Arlington Heights, IL, USA), and 6-methylmercaptopurine riboside (6-MMPr) was purchased from Millipore-Sigma (Burlington, MA, USA).

Experimental design: Suboptimal doses of FAVI, based on previous studies in this hamster model of YFV [1], were selected to evaluate the potentiation effect of 6-MMPr. For tolerability studies, 3 uninfected hamsters per group were treated with twice-daily (bid) intraperitoneal (i.p.) injections of 6-MMPr for 7 days at doses of 20 or 40 mg/kg/d, either alone or in combination with bid oral FAVI at doses of 100 or 200 mg/kg/d for 7 days. The combination groups were compared with those treated with monotherapy 6-MMPr (40 mg/kg/d) or a vehicle. For challenge studies, 10 hamsters per group were challenged with YFV via bilateral IP injections. Sham infection was performed with vehicle used for virus dilutions and was inoculated in the same way as the virus. Animals were treated i.p. bid with treatment (same doses as above) initiated 4 h prior to infection. Combinations of FAVI and 6-MMPr were compared with monotherapy and vehicle treatment. In a follow-up study, 15 hamsters per group were used to evaluate the effect of treatment with 100 mg/kg/d of FAVI combined with 20 mg/kg/d of 6-MMPr when administered beginning as late as 2 days post-virus infection (dpi). The combination groups were compared with those treated with monotherapy of either compound or vehicle. Hamsters were monitored for mortality from 0 to 21 dpi. Individual weights were recorded at 0 dpi and every other day from 3 to 15 dpi. Serum was collected on 4 and 6 dpi for analysis of virus titer and ALT. In the second study, liver and spleen were obtained from a cohort of 5 animals per group for virus titration. A strategy to minimize confounders was not used, although technicians were blinded to the nature of the treatments, with each cage receiving a group number with no identifiers on the cage or treatment bottle to reveal the treatment. The corresponding author was aware of group allocations.

Infectious cell culture assay: Virus titer in tissue or serum was assayed using an infectious cell culture assay. Briefly, serial dilutions of tissue homogenate or serum were added to Vero cells. The cytopathic effect (CPE) was used to identify the endpoint of infection. The 50% cell culture infectious doses (CCID_50_) per milliliter of plasma or gram of tissue was calculated using four replicate columns of tissue dilutions.

Serum aminotransferase assays: Serum was collected from animals 6 days post-virus inoculation (dpi) via ocular sinus bleed. Alanine aminotransferase (ALT) levels were quantified using a colorimetric reagent kit (Teco Diagnostics, Anaheim, CA, USA), which was adapted for use in a 96-well plate format. Briefly, 50 µL of substrate was used per well, and 10 µL of sample was added at timed intervals. After incubation at 37 °C, 50 µL of color reagent was added to each sample according to the same time intervals and incubated for 10 min. A volume of 200 µL of color developer was added and incubated for 5 min to stop the reaction, and the plate was read on a spectrophotometer. The concentration of ALT was then calculated per the manufacturer’s instructions by dividing the absorbance reading of the unknown plate by that of the calibrator and multiplying it by 70 to obtain the amount of international units (IU)/L.

Statistical analysis: Survival data were analyzed using Wilcoxon log-rank survival analysis, and all other statistical analyses were performed using one-way ANOVA via Dunnett multiple comparison (Prism 10, GraphPad Software, Inc., San Diego, CA, USA). Exclusion criteria were not set, and no samples were excluded from the analysis.

Ethics regulation of laboratory animals: Animal studies were conducted in accordance with the guidelines of the Institutional Animal Care and Use Committee of Utah State University (Protocol #10010). The work was performed under BSL-3 conditions in the AAALAC-accredited Laboratory Animal Research Center of Utah State University.

## 3. Results

The tolerability and efficacy of the FAVI/6-MMPr drug combination in a YFV hamster model, either as a prophylactic treatment or a 2-day-delayed treatment, is presented below.

### 3.1. Tolerablity

**Figure 3:**
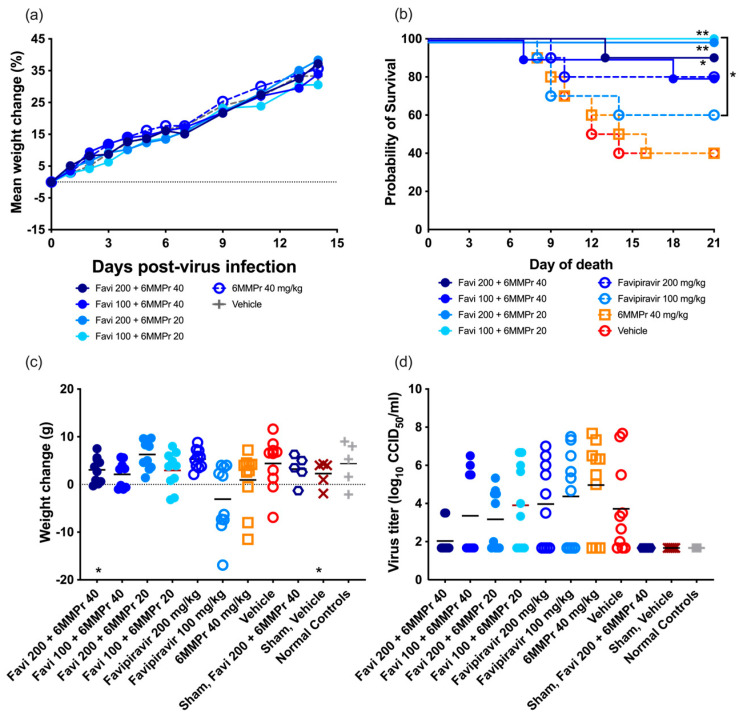
The effect of the combination of FAVI and 6-MMPr on (**a**) % weight change over time in uninfected animals, (**b**) survival, (**c**) weight change between 4 and 7 dpi, and (**d**) viremia titers at 6 dpi (*n* = 15/infected, *n* = 10/uninfected, *n* = 5/normal control groups) of infected hamsters treated with combination or monotherapy of FAVI and 6-MMPr administered beginning 4 h prior to challenge with YFV (** *p* < 0.01, * *p* < 0.05, when compared with vehicle). *n* = 10/infected group, *n* = 5/uninfected group.

A tolerability study was conducted to ensure the selected doses of 200 or 100 mg/kg/d of FAVI and 20 or 40 mg/kg/d of 6-MMPr were well tolerated when given in combination. No mortality was observed. All groups of animals treated with the combination of both compounds increased in average weight regardless of dose (Figure 3a). We selected these doses for use in the antiviral study.

### 3.2. Efficacy

#### 3.2.1. Efficacy of Prophylactic Treatment

Combinations of various doses of FAVI and 6-MMPr were tested in a hamster model of YFV for efficacy as compared with monotherapy. Oral bid treatment with FAVI, at doses of 200 or 100 mg/kg/d, was administered alone or in combination with bid i.p. treatment of 40 or 20 mg/kg/d of 6-MMPr.

Neither dose of FAVI was effective in significantly improving survival when administered as a monotherapy as compared with placebo, although a trend towards improvement was observed after treatment with 200 mg/kg/d. Treatment of infected hamsters with 40 mg/kg/d of 6-MMPr alone did not improve survival, and the mortality curve of this group was similar to that of vehicle-treated animals (Figure 3b). However, a combinatorial effect of FAVI with 6-MMPr was observed with significant improvement in survival of YFV-infected hamsters treated with a combination of FAVI and 6-MMPr as compared with vehicle (Figure 3b). Interestingly, the combination of either dose of FAVI with 20 mg/kg/d of 6-MMPr resulted in complete survival of infected hamsters.

Weight change between 4 and 7 dpi was not significantly improved with any treatment, although a trend towards improvement was observed in animals treated with combinations of 100 mg/kg/d of FAVI and 20 or 40 mg/kg/d of 6-MMPr as compared with monotherapy (Figure 3c). Treatment with 200 mg/kg/d of FAVI resulted in weight gain, while treatment with the lower dose of 100 mg/kg/d resulted in around half of the animals losing weight (Figure 3c), which was consistent with survival data. Animals treated with vehicle had an overall positive weight gain between 4 and 7 dpi, which was not consistent with previous studies.

Serum was collected at 6 dpi for the evaluation of the effect of combination treatment on viremia. None of the groups had significantly different viremia titers when compared with the placebo treatment (Figure 3d), although there was a trend towards reduction in groups treated with FAVI and 6-MMPr when compared with monotherapy or vehicle treatment. Alanine aminotransferase levels in serum, which is a marker for liver damage in this model, were evaluated in serum collected 6 dpi, but relatively low levels were detected, and no significant differences were observed between groups.

#### 3.2.2. Efficacy of 2-Day-Post-Infection (2dpi) Treatment

The combination of FAVI and 6-MMPr was tested in a hamster model of YFV when given at the time of virus challenge (−4 h) or at 2 dpi to determine the combinatorial effect of 20 mg/kg/d of 6-MMPr with 100 mg/kg/d of FAVI. Hamsters were monitored for survival, weight change, serum ALT, viremia, and virus titer in the liver and spleen to determine the effect of treatment.

**Figure 4:**
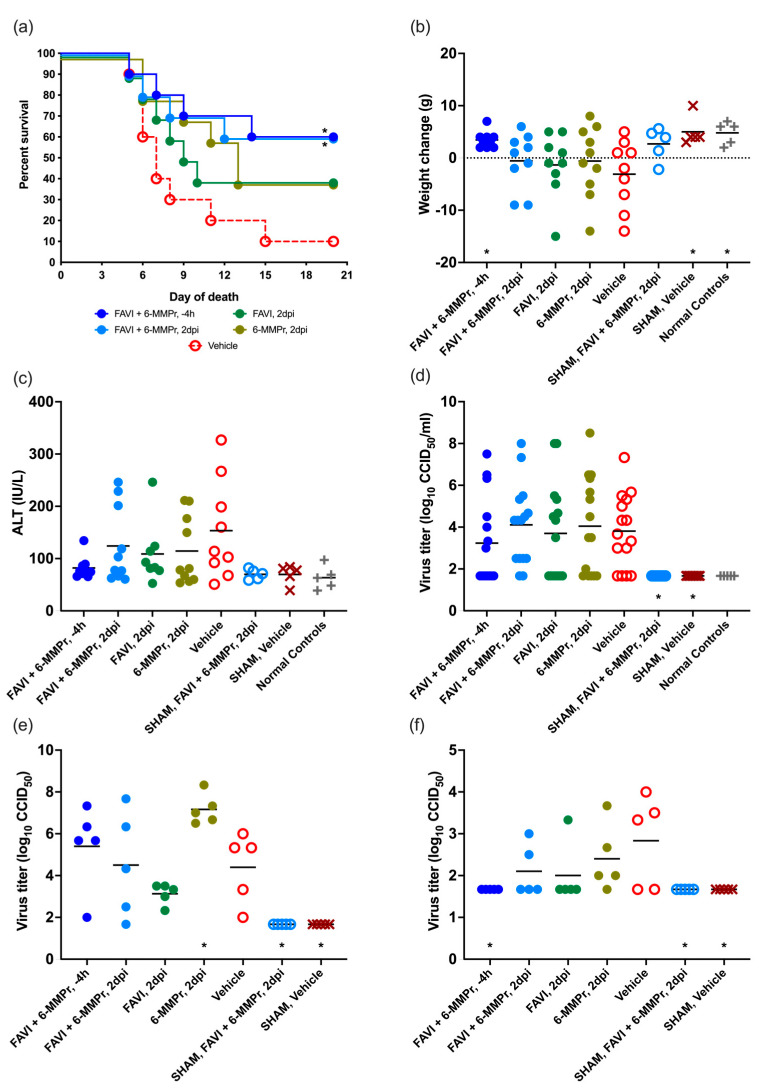
Comparison of combination of 100 mg/kg/d of FAVI with 20 mg/kg/d of 6-MMPr administered beginning 4 h before or 2 days after challenge with YFV in terms of (**a**) survival (*n* = 10/infected group, *n* = 5/uninfected group), (**b**) weight change between 4 and 6 dpi (*n* = 9–10/infected group, *n* = 5/uninfected group), (**c**) serum ALT at 6 dpi (*n* = 9–10/infected group, *n* = 5/uninfected group), and virus titer from (**d**) serum (*n* = 15/infected group, *n* = 10/uninfected group, *n* = 5/normal control group), (**e**) liver (*n* = 5/infected or uninfected group), and (**f**) spleen (*n* = 5/infected or uninfected group) collected 4 dpi (* *p* < 0.05, when compared with vehicle).

Treatment with FAVI or 6-MMPr alone did not significantly improve survival rates of infected hamsters when compared with the placebo, although a 40% survival rate was observed in these groups, while only a 10% survival rate was observed in vehicle-treated hamsters infected with YFV (Figure 4a). Treatment with a combination of FAVI and 6-MMPr resulted in a significant (*p* < 0.05) improvement in survival of treated animals when compared with vehicle treatment, even when treatment was administered 2 dpi (Figure 4a). The survival rate of animals treated with this combination was not significantly different than monotherapy despite a survival rate of 60% in the combination group.

Combination therapy with FAVI and 6-MMPr resulted in significant improvement in weight change between 4 and 6 dpi when treatment was initiated at the time of virus challenge, but not when treatment was initiated after challenge (Figure 4b). This improvement was similar to that of sham-infected and normal control hamsters.

Serum ALT was measured from serum collected on 6 dpi. There was no significant difference in mean levels of ALT, although there was a trend towards reduced levels with a combination of FAVI with 6-MMPr when treatment was initiated 4 h prior to virus challenge (Figure 4c). The variable range in ALT values collected from vehicle-treated animals precluded statistical power in the analysis of this parameter, although this group had the highest average level of serum ALT when compared with all other treatment groups.

Viremia was quantified from serum collected 4 dpi. No significant differences were observed between YFV-infected hamsters treated with combinations or monotherapies of FAVI and 6-MMPr (Figure 4d). The variability in 4 dpi serum titers was high and was similar for all infected groups. No virus was detected in sham-infected or normal control animals. Liver and spleen samples were collected from a cohort of animals at 6 dpi for viral titration. There was no effect of treatment on virus titer in the liver, although there was a significantly elevated average titer in animals treated with 6-MMPr monotherapy when compared with the placebo (Figure 4e). Spleen titers were significantly (*p* < 0.05) reduced when combination therapy with FAVI and 6-MMPr was initiated at −4 h, but not when treatment was initiated at 2 dpi (Figure 4f).

## 4. Discussion

While the activity of FAVI has been demonstrated in a hamster model of YFV [1], an animal model that demonstrates close similarities with YF disease progression in humans, progress towards clinical development has been stalled as a result of mixed results [6,7,8,9]. FAVI has broad-spectrum antiviral activity against many viruses and potential to treat different viral infections [1,2,5,14]. However, the modest efficacy of FAVI in several clinical trials against a variety of viruses [6,7,8,9], despite the use of high doses, underscores the need for strategies to optimize FAVI’s efficacy. The poor conversion of FAVI to its active form was identified previously as a possible source for its limited efficacy [10]. Herein, we demonstrate synergistic efficacy of a combination of FAVI with a *de-novo*-purine-nucleotide biosynthesis PPAT inhibitor 6-MMPr in a hamster model of YFV, confirming previous results of this synergy in vitro [11]. The translation of this strategy from in vitro to in vivo was not obvious since this strategy relies on the inhibition of a host enzyme (PPAT). However, the fact that the *de-novo*-nucleotide biosynthesis of purines and pyrimidines is upregulated in infected cells [15] suggested that a selective targeting of virally infected cells was possible without side effects. We therefore performed an *in vivo* evaluation of our drug combination strategy to demonstrate the feasibility of this approach.

In a previous study demonstrating the efficacy of FAVI in hamsters infected with YFV, a dose of 400 mg/kg/d, and to a lesser extent 200 mg/kg/d, was shown to be effective in prophylactic and therapeutic treatment regimens in hamsters, while a lower dose of 100 mg/kg/d did not significantly protect hamsters from morbidity and mortality [1]. This effective dose regimen is consistent with FAVI dosing in clinical trials, which generally use a loading dose of ~50 mg/kg/d followed by a maintenance dose of 20 mg/kg/d (hamster dose equivalents of 370 and 150 mg/kg/d, respectively, by surface area conversion) administered for two weeks [8]. The lack of activity following treatment with 100 mg/kg/d of FAVI was confirmed in the present study, which did not improve any parameters of disease.

Treatment with 6-MMPr at doses of 20 or 40 mg/kg/d alone or in combination with FAVI was well tolerated in uninfected hamsters. Treatment with 6-MMPr has shown antiviral efficacy in cell culture studies [11,16,17,18], but we did not observe any measurable antiviral effect with 6-MMPr monotherapy in hamsters infected with YFV. A combination of a suboptimal dose of FAVI with 6-MMPr significantly improved survival of hamsters infected with YFV after prophylactic or therapeutic administration, supporting previous studies in cell culture [11].

## 5. Conclusions

The successful use of FAVI and 6-MMPr, both prophylactically and therapeutically, to safely and effectively treat YFV in an animal model represents a significant step toward developing therapies for YFV and potentially other viral infections. The advantages of the synergistic combination of FAVI and a *de-novo*-nucleotide biosynthesis inhibitor include, improved efficacy, lower doses and possibly, reduced toxicity, higher compliance, a greater resilience to the development of drug-resistance. It is anticipated that future studies with these treatments, or modified compounds with more robust activity, will identify improved options for the treatment of yellow fever.

## Data Availability

All data will be made available upon request.

## Abbreviations

The following abbreviations are used in this manuscript:

YFV	yellow fever virus
FAVI	favipiravir
6-MMPr	6-methylmercaptopurine riboside
PRPP	phosphoribosyl pyrophosphate
HGPRT	hypoxanthine–guanine phosphoribosyltransferase
dpi	days post-virus inoculation
ALT	alanine aminotransferase
CID_50_	50% cell culture infectious doses
